# The EQ-5D-5L index as a measure of health-related quality of life in type 2 diabetes: a systematic review and meta-analysis

**DOI:** 10.3389/fendo.2026.1846954

**Published:** 2026-08-31

**Authors:** Adrianapia Maria Lamedica, Elena Tortato, Federica Turchi, Maria Paola Luconi, Luca Antognoli, Angelica Giuliani, Fabiola Olivieri, Liana Spazzafumo, Anna Rita Bonfigli

**Affiliations:** 1 Scientific Direction, IRCCS INRCA, Ancona, Italy; 2 Diabetology Unit, IRCCS INRCA, Ancona, Italy; 3 Clinic of Laboratory and Precision Medicine, IRCCS INRCA, Ancona, Italy; 4 Department of Clinical and Molecular Sciences (DISCLIMO), Università Politecnica delle Marche, Ancona, Italy; 5 Biogerontology Unit, Geroscience Department, IRCCS INRCA, Ancona, Italy

**Keywords:** comorbidity burden, diabetic complications, EQ-5D-5L, EQ-5D-5L index, HRQOL, type 2 diabetes

## Abstract

**Introduction:**

Type 2 diabetes (T2D) substantially impairs health-related quality of life (HRQoL). Although the EQ-5D-5L index is a validated instrument for measuring health utilities in this patient population, it remains underutilized in clinical practice. This review synthesizes current evidence regarding its cross-sectional sensitivity to the clinical burden of T2D.

**Methods:**

Following PRISMA 2020 guidelines, PubMed, Embase, Scopus, Web of Science, and ScienceDirect were searched for cross-sectional studies published from January 2000 to September 2025 reporting EQ-5D-5L index values in community-dwelling adults with T2D.

**Results:**

Forty-two studies were included, encompassing 32, 782 patients with T2D. The evidence shows significant sample size heterogeneity (ranging from 93 to 13, 966 subjects) and a stark geographic imbalance, with 69.05% of studies conducted in Asia. Results indicate that while uncomplicated T2D has a limited impact on HRQoL, the accumulation of comorbidities drastically reduces patient well-being. Specifically, cross-sectional data show a consistent inverse correlation between clinical burden and EQ-5D-5L index scores; the lowest utility values were observed in patients with diabetic complications and comorbidities, as well as those characterized by adverse socio-demographic factors, including advanced age, low education, and poverty. The substantial heterogeneity observed across the included studies was further confirmed and quantified by the meta-analysis.

**Conclusion:**

While the EQ-5D-5L index is a reliable tool for cross-sectional HRQoL assessment in T2D, longitudinal studies are needed to determine if declining index values prospectively predict diabetes complications. Ultimately, routine clinical integration of the EQ-5D-5L holds promise for personalizing care and optimizing long-term quality of life in aging patients.

**Systematic review registration:**

OSF, identifier DOI 10.17605/OSF.IO/BQYR7.

## 1 Introduction

T2D is a chronic condition with a rising global prevalence, particularly among older adults, where it poses complex clinical and functional challenges (1, 2). The growing number of individuals affected by T2D substantially increases the burden on healthcare systems, highlighting the need to assess not only metabolic outcomes but also the broader impact of the disease on patients’ HRQoL (3–6) HRQoL is a multidimensional construct encompassing physical, psychological, and social domains, reflecting how disease and treatments shape individuals’ perceived health status and everyday functioning (7, 8). In recent years, HRQoL has been increasingly recognized as a key outcome in T2D management guidelines, aligning with a patient-centered care paradigm that prioritizes well-being and functional autonomy (9). Among generic instruments for measuring HRQoL, the EuroQol 5-Dimension (EQ-5D) questionnaire is one of the most widely used tools due to its simplicity, robustness, and applicability across diverse health conditions (10, 11). Developed to provide a standardized system for describing and valuing health states, the EQ-5D supports both clinical decision-making and health economic evaluations (12).

The introduction of the EQ-5D-5L has enhanced measurement precision by expanding response options from three to five levels, thereby mitigating ceiling effects and improving sensitivity to clinically meaningful fluctuations in chronic conditions (13–16). Central to the instrument is the EQ-5D-5L Index, a EQ-5D-5L index ranging from 0 (representing death) to 1 (full health), with some value sets allowing for scores below 0 to represent states perceived as worse than death, facilitating standardized comparisons across populations (17–19). Evidence consistently demonstrates the impact of T2D on HRQoL. Extensive population-based research has consistently shown that individuals with T2D present significantly lower EQ-5D-5L scores than non-diabetic counterparts, with further decline associated with increasing diabetes-related comorbidities (20). Despite its strengths, some limitations of the EQ-5D-5L, such as residual ceiling effects in patients with mild disease, remain documented, although its psychometric validity in diabetic populations continues to be confirmed (21).

While the EQ-5D-5L has gained substantial global traction in research settings, its systematic integration into routine T2D clinical registries remains fragmented, with significant heterogeneity persisting in its application and standardized reporting. In particular, the EQ-5D-5L index is underutilized in clinical practice as a summary measure of HRQoL in T2D patients. A comprehensive synthesis of this different literature is therefore timely to highlight the instrument’s relevance in capturing the complex clinical features of diabetes. This review provides an up-to-date synthesis of the evidence to demonstrate the sensitivity of EQ-5D-5L index in assessing HRQoL among individuals with T2D, exploring factors influencing its value and discussing its implications for clinical practice.

## 2 Methods

### 2.1 Search strategy and selection criteria

This systematic review was conducted in accordance with the Preferred Reporting Items for Systematic Reviews and Meta-Analyses (PRISMA 2020) guidelines. The review protocol was retrospectively registered in the Open Science Framework (OSF) database (Registration number: DOI 10.17605/OSF.IO/BQYR7). The literature search was conducted systematically across major scientific databases, including PubMed, Embase, Scopus, Web of Science, and ScienceDirect. To ensure maximum sensitivity and avoid missing relevant literature on patient-reported outcomes (PROs), we adopted a broad-spectrum search approach. While the core search syntax targeted the combination of (“EQ-5D” OR “EQ-5D Index” OR “EQ-5D-5L”) AND (“Diabetes” OR “Type 2 Diabetes” OR “T2D”), the specific Boolean operators, controlled vocabularies (MeSH/Emtree terms), and field tags (such as the ‘ALL’ field in Web of Science) were tailored to each database. Full, reproducible details of these database-specific search strategies are provided in **Supplementary File S1**. Publications published from January 2000 up to September 2025 were considered for inclusion. Eligible studies were required to meet the following inclusion criteria: adult patients (≥ 18 years) diagnosed with T2D; a cross-sectional observational design; and a focus on community-dwelling patients. Additionally, studies had to administer the EQ-5D-5L questionnaire and report the corresponding EQ-5D-5L Index value. Conversely, studies were excluded if they evaluated only Type 1 diabetes, prediabetes, or gestational diabetes, or were not written in English. Studies that failed to report the EQ-5D-5L index value were also excluded.

### 2.2 Selection of studies

Database searches were performed independently by two authors. The initial screening phase involved title and abstract review, with subsequent exclusion of duplicate records. Eligible studies were then identified through a meticulous full-text analysis based on the inclusion/exclusion criteria. Disagreements in the selection phase were settled by an independent third party.

### 2.3 Data extraction

Data extraction was conducted in duplicate by two independent reviewers. The parameters collected included authorship, recruitment location, patient demographics (mean/median age), total enrollment, study population, and design, along with the reported EQ-5D-5L index values. The primary outcome measures were the EQ-5D-5L index scores; these were extracted and presented as means with standard deviations (SD), medians with interquartile ranges (IQR), ranges (min-max), or 95% confidence intervals (95% CI), depending on the reporting format of the original studies. A comparative analysis of the extracted data was performed to maintain high reliability and resolve any inconsistencies. Data were extracted as reported in the original studies; in cases of missing or unclear information, no assumptions or statistical imputations were made, and the data were marked as not reported.

Data extraction was restricted to baseline characteristics of the diabetic cohorts. Control group data and longitudinal follow-up outcomes were excluded to maintain a consistent cross-sectional perspective across all included evidence.

### 2.4 Quality assessment

The methodological quality of the included studies was assessed by two independent reviewers using the Joanna Briggs Institute (JBI) critical appraisal checklist for cross-sectional studies (22). Any disagreements were resolved through discussion or by consulting a third reviewer.

Overall, the 42 included studies demonstrated consistently high methodological quality (**Supplementary File S3**). The main reporting gap was an ‘Unclear’ score for Item 1 (Criteria for Inclusion) in 10 studies (23.8%). Nevertheless, all 42 papers achieved a sufficient quality threshold for inclusion, confirming the methodological rigor of the included studies (**Supplementary File S3**).

### 2.5 The EQ-5D-5L questionnaire: structure and composition

The EQ-5D-5L is a generic, standardized instrument widely used to assess HRQoL in both clinical and health economic evaluations. Developed by the EuroQol Group as an enhanced version of the EQ-5D-3L, it was specifically designed to improve sensitivity in detecting subtle variations in health status and to reduce the well-known ceiling effect observed in the earlier version (13). The questionnaire consists of two components. The first is the Descriptive System, which assesses the individual’s current health across five dimensions Mobility, Self-care, Usual Activities, Pain/Discomfort, and Anxiety/Depression. Each dimension is rated on five levels of severity (from “no problems” to “extreme problems/unable to”), allowing for the classification of 3125 distinct health states, each summarized by a five-digit code. The second component is the EQ Visual Analogue Scale (EQ VAS), a 0–100 scale on which respondents rate their perceived overall health status, providing a global, subjective measure complementary to the descriptive profile. Comparative studies have demonstrated that the EQ-5D-5L outperforms the 3L version in capturing clinically meaningful changes over time, particularly in longitudinal contexts such as rehabilitation or post-surgical recovery (23). Systematic reviews further confirm that the 5L version significantly reduces ceiling effects, especially in general population samples (24). Its psychometric properties, including validity, reliability, and responsiveness, have been extensively validated across various chronic diseases and cultural settings (25). Moreover, the EQ-5D-5L has shown greater sensitivity than the 3L in detecting treatment benefits in randomized controlled trials, such as those assessing outcomes after cataract surgery (26).

### 2.6 How the EQ-5D-5L index is calculated

The EQ-5D-5L index, or EQ-5D-5L index, is a single summary score derived from the patient’s responses to the EQ-5D-5L descriptive system. It does not incorporate the EQ VAS, which instead provides a subjective global assessment of health status. The calculation of the EQ-5D-5L index is based on country-specific value sets (or “utility tariffs”), produced through preference-elicitation studies conducted on representative population samples. These valuation studies typically employ Time Trade-Off (TTO), composite TTO (cTTO), or Discrete Choice Experiments (DCE) to quantify how the general population values different EQ-5D-5L health states (27). Methodological foundations of these valuation techniques and their integration in the EQ-5D framework are described extensively by Oppe et al. (28) and by Devlin et al. (29). In countries where a national value set for the EQ-5D-5L is not yet available, mapping (or “crosswalk”) algorithms are employed to translate EQ-5D-5L health states into EQ-5D-5L indexs based on EQ-5D-3L tariffs. One of the most widely adopted interim solutions was developed by Van Hout et al. (30), providing a validated bridge between the two systems. Recent examples highlight the variability of value sets across countries. For instance, the Polish EQ-5D-5L value set was developed through a representative national study incorporating both cTTO and DCE, allowing for negative EQ-5D-5L indexs for states considered worse than death (31). In Italy, a national EQ-5D-5L value set was generated using videoconference-based valuation interviews, reflecting population-specific preferences and providing coefficients for each severity level across the five dimensions (32). Although the exact mathematical formula varies by country, value sets generally consist of: (1) a constant (or intercept), (2) individual coefficients for each severity level of each EQ-5D dimension, and (3) optional interaction terms capturing combinations of severe problems. The methodological structure and rationale for these components are explained in the EQ-VT protocol and expanded in Devlin et al. (29). Some severe or combined health states may be assigned EQ-5D-5L index below zero indicating states valued as worse than death depending on national preferences (18, 29).

The EQ-5D utility index condenses a patient’s self-reported health state into a single score, enabling straightforward comparisons across populations and interventions. It is calculated as:


$$\text{EQ-}5\text{D-}5\text{L index}=1-\sum_{i=1}^{5}w_{i}$$

where each *w_i_* represents the utility weight associated with the severity level reported by the patient for dimension i. The five dimensions are: Mobility, Self-Care, Usual Activities, Pain/Discomfort, and Anxiety/Depression. For each dimension, the patient selects a severity level ranging from 1 (“no problems”) to 5 (“extreme problems”). If level 1 is selected, the corresponding weight equals zero, leaving the index unaffected. For levels 2 through 5, weights are drawn from a country-specific value set derived from general population preference studies.

In practice, a patient’s responses generate a five-digit health state profile for example, 21342 which is matched against the national tariff to yield a single EQ-5D-5L index. A score of 1 represents perfect health; a score of 0 corresponds to a health state equivalent to death; negative values, though uncommon, reflect states considered worse than death by the general population. For the EQ-5D-5L, index values typically range from 0.281 to 1 when using the English value set, though the exact range varies by country (18, 29).

### 2.7 Statistical analysis

Studies reporting median and interquartile range, or median and range, were converted to means and SDs using standard methods (33, 34). A random-effects meta-analysis (DerSimonian-Laird estimator) was performed to pool the EQ-5D-5L index across studies. Heterogeneity was assessed using I² and Cochran’s Q test, and a 95% prediction interval was calculated. The pooled estimates and individual study results were visualized using a forest plot. Analyses were performed in R (version 4.6.0), using the metafor package (version 5.0.1).

## 3 Results

### 3.1 Literature searches

**Figure 1:**
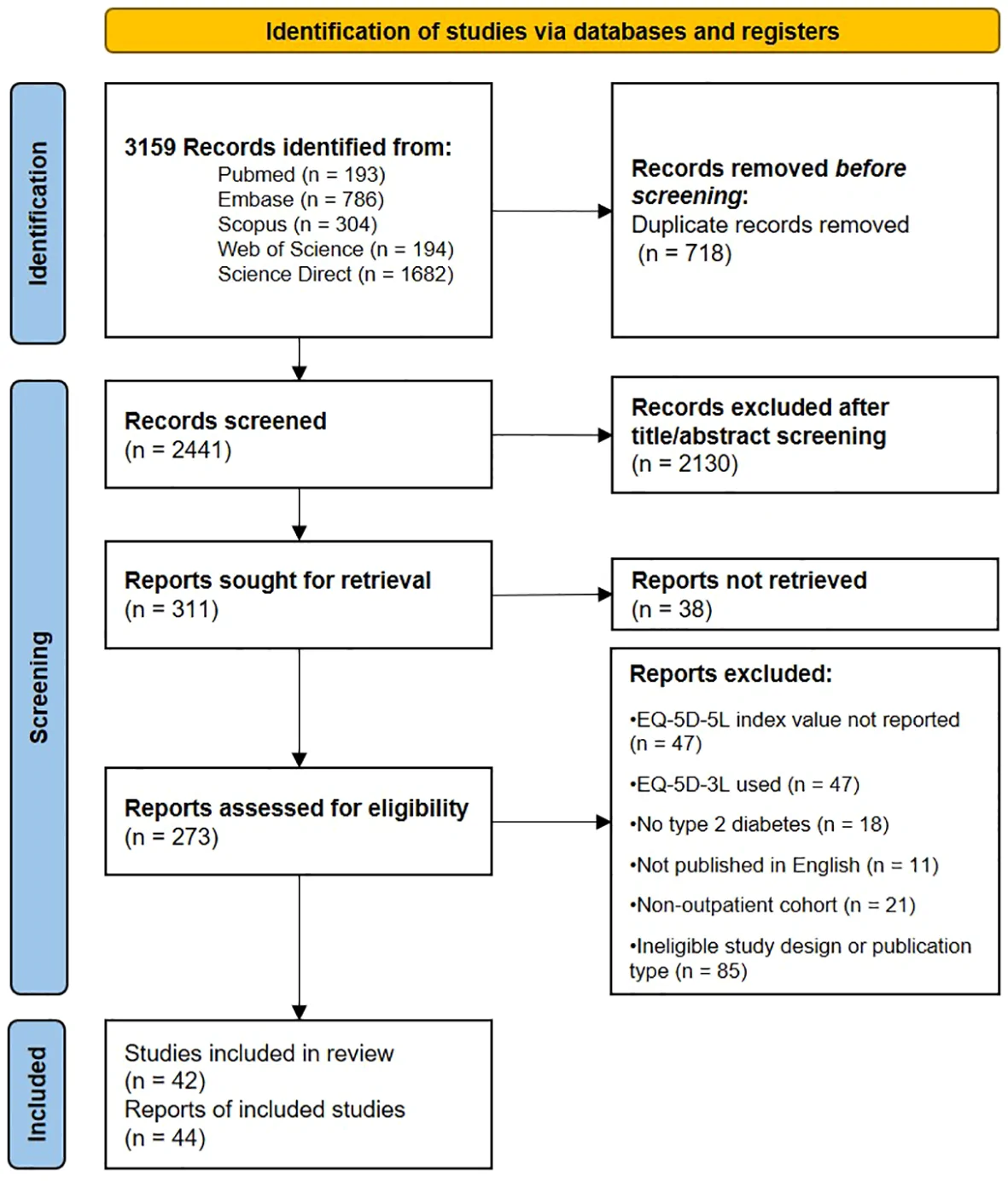
Summary of the literature search and selection.

The systematic literature search across the five selected databases yielded a total of 3, 159 records (**Figure 1**): 193 from PubMed, 786 from Embase, 304 from Scopus, 194 from Web of Science, and 1, 682 from ScienceDirect. Prior to screening, 718 duplicate records were identified and removed, leaving 2, 441 unique records for title and abstract screening. Of these, 2, 130 records were excluded as they did not meet the core objective of the review, leaving 311 reports sought for retrieval. Full-text retrieval was unsuccessful for 38 of these reports. Consequently, 273 reports were formally assessed for eligibility through a detailed full-text review. During this phase, 231 reports were excluded based on strict, predefined criteria: 47 failed to report the specific EQ-5D-5L index value, 47 utilized the older EQ-5D-3L version, 18 did not focus on T2D, and 11 were not published in English. Furthermore, in line with our focus on outpatient populations and cross-sectional observational data, 21 reports were excluded because they involved a non-outpatient cohort, and 85 were excluded due to an ineligible study design. In one case of multiple publications derived from the same patient cohort, only the most recent and clinically relevant reference was included (35). Previous papers from the same cohort were excluded as they focused purely on methodological aspects of the measurement tool (13, 36). After excluding these two papers, this rigorous selection process led to final inclusion of 42 distinct studies that met all eligibility criteria. Together, these papers provide a comprehensive overview of HRQoL measured by the EQ-5D-5L index in patients with T2D, detailing its statistical associations with relevant sociodemographic and clinical characteristics (**Supplementary Files S1**, **S3**). Despite the high methodological quality of the included studies, the uniform cross-sectional design of the 42 papers requires caution when interpreting the directionality of the observed associations.

The included studies were conducted across different geographical regions, with a strong predominance in Asia (N = 28), followed by contributions from Europe (N = 6), Africa (N = 4), and the Middle East (N = 4).

Patient age across the included studies varied significantly, although a consistent trend toward older populations was observed. Analysis of the data confirms that the overall age distribution ranges from the youngest subjects aged 18–22 years to the oldest reaching 85 years. Several studies highlighted the older demographic profile of their cohorts. For instance, O’Shea et al. (37) reported that 78% of subjects were aged 55 years or older, while Prabowo et al. (38) and Dinkova et al. (39) noted that 91.5% and 90.3% of their respective participants were aged 50 or above, a trend consistent with the chronic nature of the condition.

The sample sizes across the 42 included studies were highly heterogeneous, with the total number of subjects enrolled amounting to 32, 782 (mean sample size: 780.5 participants). The number of participants in individual studies exhibited extreme variability, ranging from a minimum of 93 subjects (39) to a maximum of 13, 966 subjects (40). This indicates that the evidence base is composed of many small-to-moderate clinical cohorts alongside a few extensive cross-sectional surveys.

HRQoL among the included patients with T2D exhibited wide variability, with EQ-5D-5L index values ranging from a minimum of 0.47 (39) to a maximum of 0.95 (41). The data suggest a common pattern with higher index scores generally associated with younger, uncomplicated cohorts, and lower scores more frequently reported in older cohorts facing advanced chronic complications.

### 3.2 Determinants of EQ-5D-5L index in T2D based on selected literature

The marked heterogeneity in EQ-5D-5L index scores underscores the diverse clinical spectrum of T2D. Within this population, HRQoL is shaped by numerous factors extending far beyond glycemic control. Multimorbidity remains a major determinant of reduced health utility; indeed, several studies from Singapore, China, and India consistently report lower EQ-5D-5L index values in patients with multiple comorbidities or longer disease duration. This cumulative burden is clearly illustrated by the compromised utility scores reported by Chandran et al. (3) (0.81 ± 0.27), Wang et al. (15) (0.79 ± 0.30), and Arifin et al. (42) (0.77 [95% CI: 0.75-0.79]) (**Table 1**). Notably, these values fall substantially below general population reference norms, suggesting a multi-dimensional erosion of health status.

**Table 1:** Summary of selected studies reporting EQ-5D-5L index values in T2D patients.

Reference (N)	Recruiting country	Patient age	Number of patients enrolled	Population	Study design	EQ-5D-5L Index values
Chandran et al. (3)	Singapore	61.1 ± 13.4**^2^**	1406	Patients with T2D with microvascular and macrovascular complications	Cross-sectional study	0.81 ± 0.27**^2^**
Wang et al. (15)	Singapore	55.5 ± 12.7**^2^**	121	Patients with T2D	Cross-sectional study	0.79 ± 0.3**^2^**
Tang et al. (43)	China	64.0 ± 9.7**^2^**	277	Patients with T2D	Cross-sectional validation study	0.90 ± 0.13**^2^**
O’Shea et al. (37)	Ireland	78% of subjects were aged 55 years or more	159	Patients with T2D	Observational cross-sectional study	0.80 (0.69 -1.00)**^3^**
Dudzińska et al. (44)	Poland	62.1 ± 9.8**^2^**	274	Patients with T2D	Observational cross-sectional study	0.79 ± 0.14**^2^**
Wong et al. (40)	China	≥ 65 years	13966	Patients with T2D	Cross-sectional study	0.84 ± 0.23**^2^**
Namdeo et al. (45)	Bangladesh	52 ± 10**^2^**	318	Patients with T2D	Cross-sectional study	0.62 ± 0.22**^2^**
Prabowo et al. (38)	Indonesia	91.5% of patients were aged ≥50 years.	236	Patients with T2D	Cross-sectional study	0.86 (0.83-0.88)**^4^**
Chen et al. (46)	Taiwan	60.1 ± 13.1**^2^**	506	Patients with T2D	Cross-sectional survey	0.88 ± 0.20**^2^**
Pham et al. (47)	Vietnam	61.5 (58 ‐67)**^3^**	214	Patients with T2D	Cross-sectional study	0.94 (0.85 – 1.00)**^3^**
Zeidalkilani et al. (48)	Palestine	60.9 ± 10.6**^2^**	158	Patients with T2D receiving hemodialysis	Multicenter cross-sectional study	0.58 (0.32 – 0.80)**^3^**
Wang et al. (49)	Singapore	56.8 ± 11.0**^2^**	729	Patients with T2D, characterized by long disease duration and a significant burden of comorbidities	Cross-sectional survey	0.84 ± 0.22**^2^**
Arifin et al. (42)	Indonesia	59.3 ± 9.7**^2^**	907	Patients with T2D	Cross-sectional study	0.77 (0.75 –0.79)**^4^**
Mihevc et al. (50)	Slovenia	72**^1^**	358	Patients with T2D	Cross-sectional survey	0.80 ± 0.27**^2^**
Fajriansyah et al. (51)	Indonesia	57.7 ± 5.6**^2^**	220	Patients with T2D	Cross-sectional study	0.54 ± 0.32**^2^**
Kamradt et al. (52)	Germany	67.8 ± 10.8**^2^**	495	Patients with T2D	Cross-sectional study	0.69 ± 0.23**^2^**
Kim et al. (53)	Korea	63.6 ± 10.6**^2^**	1338	Patients with painful diabetic neuropathy (PDN)	Cross-sectional study	PDN 0.6 ± 0.3^2*^ Non-PDN 0.8 ± 0.1^2^
Azharuddin et al. (54)	North Indian	55.29 ± 12.45**^2^**	300	Patients with T2D	Cross-sectional study	0.79 ± 0.22**^2^**
Kang et al. (55)	China	60.47 ± 9.55**^2^**	2231	Patients with T2D	Cross-sectional study	0.93 ± 0.15**^2^**
Abedini et al. (56)	Iran	58.1 ± 9.6**^2^**	300	Patients with T2D	Cross-sectional study	0.89 ± 0.13**^2^**
Ishii et al. (57)	Japan	62.6 ± 11.30**^2^**	978	Patients with T2D	Observational cross-sectional analysis	0.92 ± 0.11**^2^**
Pan et al. (35)	China	aged 18 years or older	289	Patients with T2D	Cross-sectional study	0.89 ± 0.18**^2^**
Ananthesh et al. (58)	South India	57.08 ± 11.21**^2^**	328	Patients with T2D	Cross-sectional study	0.84 ± 0.13**^2^**
Sendekie et al. (59)	Northwest Ethiopia	55.1 ± 10.7**^2^**	402	Patients with T2D	Multicentre cross-sectional study design	0.58 ± 0.17**^2^**
Tan et al. (60)	Malaysia	62 ± 11.5**^2^**	513	Patients with T2D	Cross-sectional study	0.85 ± 0.17**^2^**
Guo et al. (61)	Singapore	58.6 ± 9.5**^2^**	370	Patients with T2D	Cross-sectional study	0.9 **^1^** (without DRD) vs. 0.8**^1^** (with DRD)**
Gebremariam et al. (41)	Etiopia	64.43 ± 10.61**^2^**	360	Patients with T2D	Cross-sectional study	0.95 (0.88–0.96)**^3^**
Riandini et al. (62)	Singapore	DPN group: 64 ± 6**^2^** Non-DPN group: 60 ± 7**^2^**	160	Patients with T2D	Cross-sectional study	DPN group: 0.67 ± 0.14**^2^** ** Non-DPN group: 0.77 ± 0.16**^2^**
Zare et al. (63)	Iran	59.4 ± 20**^2^**	717	Patients with T2D	Cross-sectional study	0.75 ± 0.006**^2^**
Alshayban et al. (64)	Saudi Arabia	79% of the participants were over 50 years of age	378	Patients with T2D	Cross-sectional study	0.81 (0.65–0.94)**^3^**
Almasri et al. (65)	Saudi Arabia	55 (22-85)**^6^**	131	Patients with T2D	Cross-sectional study	Men: 0.65 (−0.37–1)**^6^** Woman: 0.59 (−0.105–1)**^6^**
Nacanabo et al. (66)	West African	80.5% of the participants were under 60 years of age	175	Patients with T2D	Cross-sectional study	0.80 ± 0.1**^2^**
Zyoud et al. (67)	Palestine	59.3 ± 11.2**^2^**	385	Patients with T2D	Cross-sectional study	0.78 ± 0.10**^2^**
Kombathula et al. (68)	India	53.73 ± 12.0**^2^**	200	Patients with T2D	Cross-sectional study	0.86 ± 0.14**^2^**
Parik et al. (69)	India	60.71 ± 11.41**^2^**	358	Patients with T2D	Cross-sectional descriptive study	0.80**^1^**
Dinkova et al. (39)	Bulgaria	90.33% of the participants were over 50 years of age	93	Patients with T2D	Cross-sectional study	Men: 0.65 ± 0.312 Women: 0.47 ± 0.312
Asrie et al. (70)	Northwest Ethiopia	49.7% of the participants were 60 years of age or younger, while 50.3% were older than 60 years	386	Patients with T2D	Cross-sectional study	0.86 (0.76-0.93)**^3^**
Marwood et al. (71)	United Kingdom	50.3 ± 9.6**^2^**	580	Patients with T2D	Cross-sectional study	0.73 ± 0.27**^2^**
Hashimoto et al. (72)	Japan	63.25 ± 10.21**^2^**	342	Patients with T2D	Cross-sectional study	no sleep disorders: 0.86 ± 0.13**^2^** sleep disorders: 0.77 ± 0.16**^2^**
Pratama et al. (73)	Indonesia	age ≥ 60	200	Patients with T2D	Cross-sectional study	Male: 0.78 ± 0.13**^2^** Female: 0.82 ± 0.14**^2^**
Endarti et al. (74)	Indonesia	< 35, 36-45, 46-55, 56–65 and > 65	178	Patients with T2D	Cross-sectional, observational study	0.87 ± 0.165**^2^**
Natasya et al. (75)	Indonesia	non-geriatric (58.3%) and geriatric (41.7%)	746	Patients with T2D	Cross-sectional study	0.74 ± 0.23**^2^**

Data are presented as mean^1^, or mean ± standard deviation (SD)^2^, median (interquartile range, IQR)^3^, or mean with 95% confidence interval (95% CI)^4^, least-square mean (SD)^5^, median (min – max) ^6^; *p<0.01; **p<0.001. DRD, Diabetes-related distress.

Data from the included cohorts reveal pronounced reductions in EQ-5D-5L index values among patients suffering from specific diabetes-related complications (**Table 1**). For instance, Zeidalkilani et al. (48) observed a median index score of 0.58 (IQR: 0.32-0.80) in patients with T2D and chronic kidney disease, while Kim et al. (76) observed a mean score of 0.75 ± 0.32 in those with painful diabetic neuropathy. Similarly, Riandini et al. (62) identified a distinct utility gap between patients with diabetic peripheral neuropathy (0.67 ± 0.14) and those without (0.77 ± 0.16). Retinopathy exhibits a similar trend, with Guo et al. (61) reporting lower index values in affected patients (0.81) compared to their complication-free counterparts (0.91). This decline becomes even more severe in cohorts specifically focusing on neuro-functional or vascular deterioration, as demonstrated by the remarkably low scores in Fajriansyah et al. (51) (0.54 ± 0.32) and Sendekie et al. (59) (0.56 ± 0.11). Ultimately, these clear variations across distinct patient samples validate the capacity of the EQ-5D-5L index to sensitively capture disease burden and differentiate degrees of health impairment in cross-sectional settings.

Beyond clinical parameters, sociodemographic and behavioral characteristics also exhibit significant associations with health utility. Almasri et al. (65) et Dinkova et al. (39) demonstrated gender-based disparities, reporting lower EQ-5D-5L index scores in women compared to men. Furthermore, sleep quality emerged as a key correlate of well-being; Hashimoto et al. (72) documented significantly lower EQ-5D-5L index scores among patients with sleep disorders (0.77 ± 0.16) compared to those without sleep disturbances (0.86 ± 0.13).

### 3.3 Statistical analysis

Although 42 studies were selected, only 41 (totaling 32, 424 patients) were included in the meta-analysis; one study was excluded because it lacked a measure of variability, reporting only a point estimate. The random-effects meta-analysis yielded a pooled mean EQ-5D-5L index of 0.787 (95% CI 0.759 – 0.815) in older adults with diabetes. Statistical heterogeneity was substantial (I² = 99.48%, τ² = 0.0083, Q (40) = 7664.61, p< 0.0001). Given this high heterogeneity, a 95% prediction interval was calculated, indicating that the true EQ-5D-5L index in a new study population could reasonably range from 0.607 to 0.968. The 95% prediction interval (0.607–0.968) was considerably wider than the confidence interval around the pooled estimate, reflecting marked variability in the true underlying EQ-5D-5L index across study populations. This high heterogeneity may be partly explained by differences in patient characteristics (e.g., age, disease duration, and presence of complications), geographic setting, and the corresponding country-specific EQ-5D-5L value sets used to derive the index, and variations in questionnaire administration methods. Given the width of the prediction interval, the pooled estimate should be interpreted with caution as a general reference value rather than being directly applicable to any specific subgroup of patients.

## 4 Discussion

The present systematic review examined 42 studies comprising a total of 32, 782 participants with T2D. Although geographically diverse, the synthesized literature exhibits a strong concentration in Asia (N = 28). Sample sizes across the evidence base varied widely, ranging from small clinical cohorts (N = 93 subjects) to large population-based database surveys (N = 13, 966 subjects) (39, 40, 53).

Furthermore, a consistent trend toward older cohorts was observed across the data, with multiple studies reporting a high proportion of patients aged 50 or 60 and older which underscores the frequent coexistence of this chronic condition with the aging process and highlights the concurrent relevance of integrated care models tailored to managing the current health status of older populations (1).

Since the final evidence base consists entirely of cross-sectional designs carries specific methodological implications must be considered. While these designs provide a valuable “snapshot” of HRQoL and allow for the identification of concurrent clinical factors, they cannot establish causality, directional pathways, or long-term longitudinal trends. Therefore, while our findings map a comprehensive network of correlations, future studies utilizing longitudinal cohorts and interventional frameworks are required to clarify how patient health and quality of life dynamically evolve over time.

**Figure 2:**
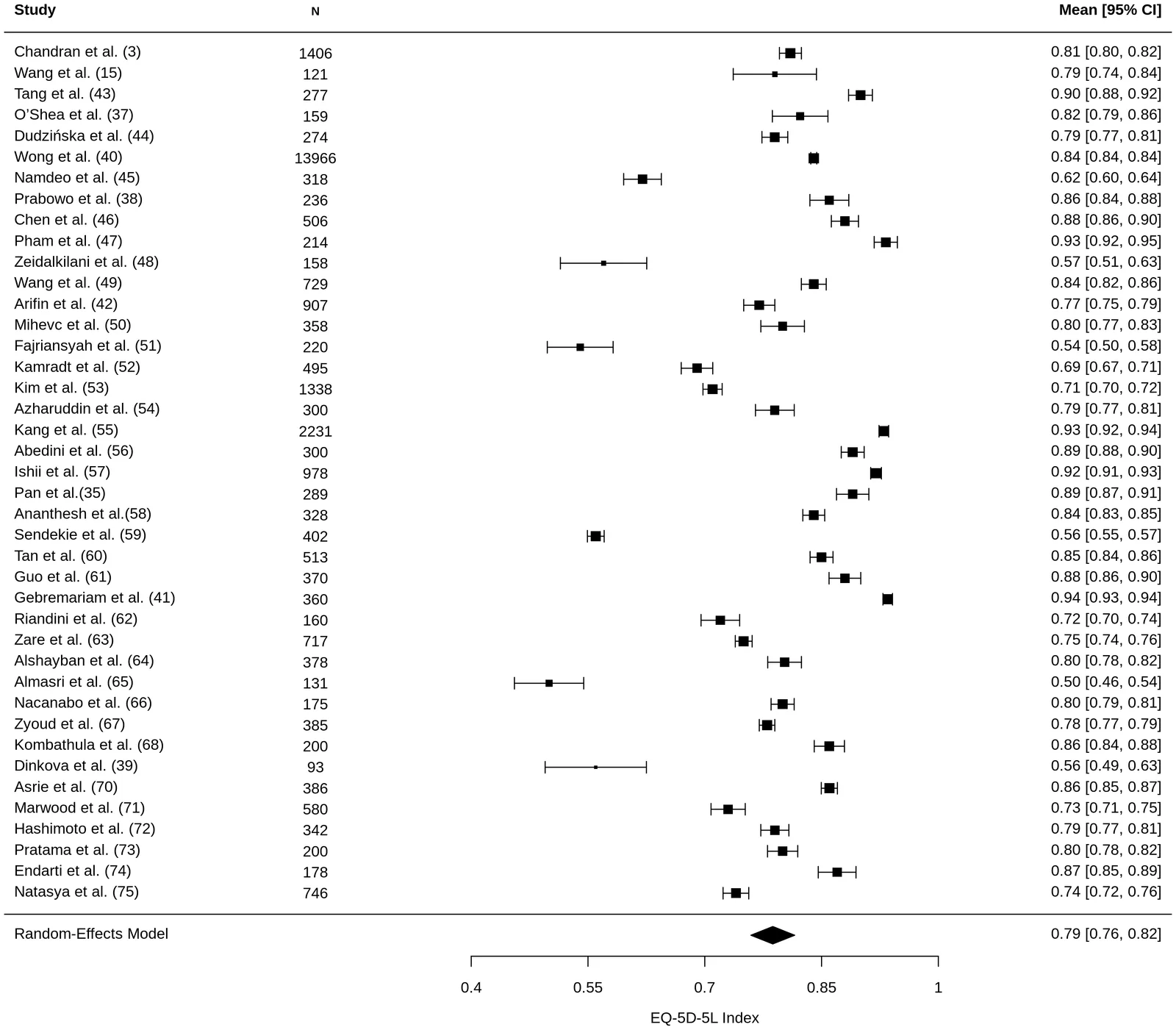
Forest plot of the meta-analysis on EQ-5D-5L index scores in patients with T2D.

Variations in scores across recruiting countries raise interesting methodological questions regarding the choice of value sets used to calculate the index. While European studies demonstrate remarkable stability with scores clustering around 0.73 - 0.80 (e.g., O’Shea et al. in Ireland: 0.80 (37); Dudzińska et al. in Poland: 0.79 (44); Marwood et al. in the UK: 0.73 (71), developing countries like Ethiopia (Sendekie et al.: 0.58 ± 0.17 (59) and Bangladesh (Namdeo et al.: 0.62 ± 0.22 (45) report much lower average utility scores compared to high-income Asian nations such as Singapore (e.g., Tang et al.: 0.90 ± 0.13 (43); Guo et al.: 0.91 (61). This discrepancy suggests that local healthcare resources and access to diabetes care profoundly shape patients’ perceived health status. Ultimately, this high degree of heterogeneity among the selected studies, spanning different economic landscapes and national value sets, is statistically validated by the meta-analysis, which demonstrates a wide and significant dispersion of health utility scores across the literature (**Figure 2**). The selected literature also includes studies conducted in countries that utilize proxy value sets to calculate the EQ-5D-5L index. Moving forward, research should continue to refine country- and population-specific EQ-5D-5L value sets to improve utility mapping accuracy (30, 31).

Literature data show a marked difference in the perception of quality of life between men and women suffering from T2D, with various studies reporting worse quality of life for women. In the study by Dinkova et al., the difference is substantial: men have a mean index of 0.65± 0.31, while women drop to 0.47 ± 0.31 (39). Almasri et al. confirm this trend, although with a smaller gap: 0.65 for men vs. 0.59 for women (65). Only the study by Pratama et al. shows an inverted trend, with women reporting a slightly higher score than men (0.82 ± 0.14 vs. 0.78 ± 0.13) (73).

A key finding of this updated synthesis is the pronounced clinical heterogeneity across the included cross-sectional cohorts. Within the selected literature, the EQ-5D-5L index scores provided valuable insights into the patients’ cross-sectional health states. Crucially, lower EQ-5D-5L scores consistently tracked alongside a higher burden of multimorbidity and secondary complications. The lowest index scores were systematically recorded among patients concurrently suffering from microvascular complications, chronic kidney disease (CKD), neuropathic pain, or advanced cardiovascular conditions (3, 48, 53). In fact, several studies have highlighted that the presence of micro- and macrovascular complications, as well as specific comorbidities, drastically reduces the EQ-5D-5L index score. Chandran et al. highlighted that HRQoL was negatively influenced by diabetes distress, lower-quality housing, older age, female sex, higher BMI, and the presence of macrovascular complications (3). In a Chinese cross-sectional study, among patients with diabetes, lower HRQoL was associated with older age, poverty, low physical activity, and the presence of comorbidities, whereas a higher educational level was positively related to HRQoL (55). Ishii et al. reported that utility values closely reflected the severity of health conditions, while minimal differences were observed when values were stratified by age, BMI, or the 7% HbA1c threshold, and no significant associations were found with clinical risk factors (57). It was reported that painful diabetic peripheral neuropathy (PDPN) impairs quality of life, measured by EQ-5D-5L index compared with non-PDPN (53). This finding is consistent with research by Riandini et al., which confirmed that patients with PDPN have a lower HRQoL than those without (62). Quality of life was lower among those with diabetes-related distress (DRD) with respect to patients without DRD (61). Diabetic patients with CKD on hemodialysis showed very low HRQoL levels (48).

Even non-somatic comorbidities influence the index. In particular, Hashimoto et al. reported a significantly lower utility score in diabetic patients suffering from sleep disorders (72). Regular cross-sectional assessments in clinical settings can therefore facilitate the early identification of patients experiencing poor health status, enabling timely, tailored supportive strategies that optimize not only metabolic targets but also functional status and mental well-being.

The above findings confirm that HRQoL is strongly associated with the presence and accumulation of comorbidities and secondary diabetic complications, as well as other key determinants such as age, sex, sleep disorders, education, and poverty. Recognizing this wide variance is crucial for clinicians and policymakers, as it underscores that HRQoL in T2D cannot be generalized as a uniform baseline, but must be strictly stratified according to the patient’s specific complication and comorbidity profile.

Overall, the EQ-5D-5L index serves as a valuable tool in diabetes research, condensing complex, multidimensional health states into a single metric suitable for cross-sectional health status assessment and health policy planning (28). While its standardized, cross-cultural validity facilitates comparative analysis across global populations, its sensitivity to highly specific, disease-focused health changes remains a subject of ongoing debate. Therefore, while the EQ-5D-5L is excellent for detecting cross-sectional variations associated with major complications, it can be usefully complemented by disease-specific instruments to ensure a comprehensive, nuanced clinical assessment (37).

Future longitudinal studies are warranted to explore whether tracking EQ-5D-5L scores over time can predict diabetic complications by detecting sub-clinical decline before clinical onset. Meanwhile, current cross-sectional data can already help clinics stratify patient burden and deliver personalized supportive care to those with compromised HRQoL. Ultimately, systematically integrating the EQ-5D-5L tool into healthcare systems holds the potential to advance patient-centered care and shape health policies aimed at optimizing quality of life in T2D.

### 4.1 Study limitations

To properly contextualize our findings, certain limitations must be addressed. Although this review draws from a large pooled sample (N = 32, 782), the clinical application of the EQ-5D-5L index in T2D remains geographically skewed. Specifically, a major geographical imbalance is evident due to the heavy predominance of studies conducted in Asia (N = 28) (40, 49). While this offers an understanding of the condition within Asian populations, the relatively low representation of other regions, such as Europe, Africa, and the Middle East, suggests that caution is warranted when generalizing these findings to different healthcare systems and socioeconomic contexts particularly given the heterogeneity of local healthcare resources across the included cohorts. Furthermore, the exclusive reliance on cross-sectional designs provides only a static snapshot of patient health, precluding the ability to infer temporal or causal relationships between T2D progression and the decline in quality of life. Additionally, a significant sample size asymmetry is present across the included studies, characterized by a wide gap in participant numbers between cohorts. This right-skewed distribution indicates that the overall evidence base is heavily weighted by a few large-scale population-based surveys, which may disproportionately influence the pooled results (40, 53). Similarly, limiting the inclusion to English-language papers introduces a potential language bias that may restrict the global representativeness of the findings. Finally, we acknowledge that the protocol for this review was registered retrospectively on the OSF database, which may theoretically introduce a risk of reporting bias.

## 5 Conclusion

This systematic review of 42 studies comprising 32, 782 participants demonstrates that the onset of diabetic complications as well as comorbidities and adverse socio-demographic factors, significantly compromises patient HRQoL. In this context, the EQ-5D-5L index serves as a reliable descriptive instrument, as its varying values consistently track the multidimensional impact of disease burden. However, the exclusively cross-sectional nature of the retrieved literature prevents the determination of causal or temporal pathways. Consequently, future research should prioritize longitudinal and interventional designs to monitor sub-clinical health decline over time and evaluate targeted multidisciplinary care models. Ultimately, integrating the systematic use of the EQ-5D-5L index into routine diabetes clinics holds the potential for advancing personalized, patient-centered care and optimizing long-term quality of life in aging populations with T2D.

## Data Availability

The original contributions presented in the study are included in the article/**Supplementary Material**. Further inquiries can be directed to the corresponding author.
