# Supplementary material for: The EQ-5D-5L index as a measure of health-related quality of life in type 2 diabetes: a systematic review and meta-analysis
Source: Front Endocrinol (Lausanne). 2026 Aug 31;17:1846954. doi: 10.3389/fendo.2026.1846954 (PMC13572114; doi:10.3389/fendo.2026.1846954)
Supplement: Supplementary file 1 [file DataSheet1.docx]

**File S1 Search strategy**

| **Pubmed** | |
| --- | --- |
| # | Query |
| 1 | "Diabetes Mellitus, Type 2"[Mesh] |
| 2 | "Type 2 Diabetes"[tiab] OR "T2DM"[tiab] |
| 3 | #1 OR #2 |
| 4 | "EQ-5D-5L"[tiab] OR "EQ5D5L"[tiab] OR "EQ-5D-5L index"[tiab] OR "EQ-5D index"[tiab] OR "utility score"[tiab] OR "utility value"[tiab] OR "utility index"[tiab] OR "health state index"[tiab] |
| 5 | #3 AND #4 |

| **Embase** | |
| --- | --- |
| # | Query |
| 1 | 'type 2 diabetes':ti,ab OR 'diabetes mellitus, type 2':ti,ab OR 't2dm':ti,ab |
| 2 | 'non insulin dependent diabetes mellitus'/exp |
| 3 | #1 OR #2 |
| 4 | 'eq-5d-5l':ti,ab OR 'eq5d5l':ti,ab OR 'eq-5d-5l index':ti,ab OR 'eq-5d index':ti,ab OR 'utility score':ti,ab OR 'utility value':ti,ab OR 'utility index':ti,ab OR 'health state index':ti,ab |
| 5 | 'euroqol'/exp |
| 6 | #4 OR #5 |
| 7 | #3 AND #6 |

| **Scopus** | |
| --- | --- |
| # | Query |
| 1 | TITLE-ABS-KEY ("Diabetes Mellitus, Type 2" OR "Type 2 Diabetes" OR "T2DM") |
| 2 | TITLE-ABS-KEY ("EQ-5D-5L" OR "EQ5D5L" OR "EQ-5D-5L index" OR "EQ-5D index" OR "utility score" OR "utility value" OR "utility index" OR "health state index") |
| 3 | #1 AND #2 |

| **Web of Science** | |
| --- | --- |
| # | Query |
| 1 | ALL=(("Diabetes Mellitus, Type 2" OR "Type 2 Diabetes" OR T2DM)) |
| 2 | ALL=(("EQ-5D-5L" OR EQ5D5L OR "EQ-5D-5L index" OR "EQ-5D index" OR "utility score" OR "utility value" OR "utility index" OR "health state index")) |
| 3 | #1 AND #2 |

| **Science Direct** | |
| --- | --- |
| # | Query |
| 1 | ("Type 2 Diabetes" OR T2DM) AND ("EQ-5D-5L index" OR "EQ-5D" OR "utility score") |
