# Supplementary material for: The EQ-5D-5L index as a measure of health-related quality of life in type 2 diabetes: a systematic review and meta-analysis
Source: Front Endocrinol (Lausanne). 2026 Aug 31;17:1846954. doi: 10.3389/fendo.2026.1846954 (PMC13572114; doi:10.3389/fendo.2026.1846954)
Supplement: Supplementary file 3 [file Table1.docx]

Supplementary Material

# 1 Supplementary Data

Table S1 - Assessment of methodological quality according to the checklist proposed by Joanna Briggs Institute for cross-sectional studies.

| Ref N. | 1.Criteria for inclusion in the sample? | 2. Study subjects and the setting described? | 3.Exposure measured in a valid and reliable way? | 4. Objective, standard criteria used for measurement of the condition | 5.Confounding factors identified? | 6. Strategies to deal with confounding factors stated? | 7.Outcomes measured in a valid and reliable way? | 8.Appropriate statistical analysis use? | Overall appraisal |
| --- | --- | --- | --- | --- | --- | --- | --- | --- | --- |
| Chandran et al. (3) | Yes | Yes | Yes | Yes | Yes | Yes | Yes | Yes | Include |
| Wang et al. (15) | Yes | Yes | Yes | Yes | Yes | Yes | Yes | Yes | Include |
| Tang et al. (52) | Yes | Yes | Yes | Yes | Yes | Yes | Yes | Yes | Include |
| O’Shea et al. (37) | Yes | Yes | Yes | Yes | Yes | Yes | Yes | Yes | Include |
| Dudzińska et al. (53) | Yes | Yes | Yes | Yes | Yes | Yes | Yes | Yes | Include |
| Wong et al. (40) | Unclear | Yes | NA | Yes | Yes | Yes | NA | Yes | Include |
| Namdeo et al. (54) | Yes | Yes | Yes | Yes | Yes | Yes | Yes | Yes | Include |
| Prabowo et al. (38) | Unclear | Yes | Yes | NA | Yes | Yes | Yes | Yes | Include |
| Chen et al. (55) | Yes | Yes | Yes | Yes | Yes | Yes | Yes | Yes | Include |
| Pham et al. (56) | Yes | Yes | Yes | Yes | Yes | Yes | Yes | Yes | Include |
| Zeidalkilani et al. (43) | Yes | Yes | Yes | Yes | Yes | Yes | Yes | Yes | Include |
| Wang et al. (57) | Yes | Yes | Yes | Yes | Yes | Yes | Yes | Yes | Include |
| Arifin et al. (42) | Yes | Yes | Yes | Yes | Yes | Yes | Yes | Yes | Include |
| Mihevc et al. (58) | Yes | Yes | Yes | Yes | Yes | Yes | Yes | Yes | Include |
| Fajriansyah et al. (47) | Yes | Yes | NA | Yes | Yes | NA | Yes | Yes | Include |
| Kamradt et al. (59) | Yes | Yes | Yes | Yes | Yes | Yes | Yes | Yes | Include |
| Kim et al. (51) | Yes | Yes | Yes | Yes | Yes | Yes | Yes | Yes | Include |
| Azharuddin et al. (60) | Yes | Yes | Yes | Yes | Yes | Yes | Yes | Yes | Include |
| Kang et al. (61) | Unclear | Yes | Yes | Yes | Yes | Yes | Yes | Yes | Include |
| Abedini et al. (62) | Unclear | Yes | Yes | Yes | Yes | Yes | Yes | Yes | Include |
| Ishii et al. (63) | Yes | Yes | Yes | Yes | Yes | Yes | Yes | Yes | Include |
| Pan et al. (35) | Yes | Yes | Yes | Yes | Yes | Yes | Yes | Yes | Include |
| Ananthesh et al. (64) | Yes | Yes | Yes | Yes | Yes | Yes | Yes | Yes | Include |
| Sendekie et al. (48) | Yes | Yes | Yes | Yes | Yes | Yes | Yes | Yes | Include |
| Tan et al. (65) | Yes | Yes | Yes | Yes | Yes | Yes | Yes | Yes | Include |
| Guo et al. (46) | Yes | Yes | Yes | Yes | Yes | Yes | Yes | Yes | Include |
| Gebremariam et al. (41) | Unclear | Yes | Yes | Yes | Yes | Yes | Yes | Yes | Include |
| Riandini et al. (45) | Yes | Yes | Yes | Yes | Yes | Yes | Yes | Yes | Include |
| Zare et al. (66) | Unclear | Yes | Yes | Yes | Yes | Yes | Yes | Yes | Include |
| Alshayban et al. (67) | Yes | Yes | Yes | Yes | Yes | Yes | Yes | Yes | Include |
| Almasri et al. (49) | Unclear | Yes | Yes | Yes | Yes | Yes | Yes | Yes | Include |
| Nacanabo et al. (68) | Yes | Yes | Yes | Yes | Yes | Yes | Yes | Yes | Include |
| Zyoud et al. (69) | Yes | Yes | Yes | Yes | Yes | Yes | Yes | Yes | Include |
| Kombathula et al. (70) | Yes | Yes | Yes | Yes | Yes | Yes | Yes | Yes | Include |
| Parik et al. (71) | Yes | Yes | Yes | Yes | Yes | Yes | Yes | Yes | Include |
| Dinkova et al. (39) | Yes | Yes | Yes | Yes | Yes | No | Yes | No | Include |
| Asrie et al. (72) | Yes | Yes | Yes | Yes | Yes | Yes | Yes | Yes | Include |
| Marwood et al. (73) | Yes | Yes | Yes | Yes | Yes | Yes | Yes | Yes | Include |
| Hashimoto et al. (50) | Unclear | Yes | Yes | Yes | Yes | Yes | Yes | Yes | Include |
| Pratama et al. (74) | Yes | Yes | Yes | Yes | Yes | Yes | Yes | Yes | Include |
| Endarti et al. (75) | Unclear | Yes | Yes | Yes | Yes | Yes | Yes | Yes | Include |
| Natasya et al. (76) | Yes | Yes | Yes | Yes | Yes | Yes | Yes | Yes | Include |
